# Prenatal whole exome sequencing detects a new homozygous fukutin *(FKTN)* mutation in a fetus with an ultrasound suspicion of familial Dandy–Walker malformation

**DOI:** 10.1002/mgg3.1054

**Published:** 2019-11-22

**Authors:** Alice Traversa, Silvia Bernardo, Alessandro Paiardini, Agnese Giovannetti, Enrica Marchionni, Maria Luce Genovesi, Daniele Guadagnolo, Barbara Torres, Stefano Paolacci, Laura Bernardini, Tommaso Mazza, Massimo Carella, Viviana Caputo, Antonio Pizzuti

**Affiliations:** ^1^ Fondazione IRCCS Casa Sollievo della Sofferenza Laboratory of Clinical Genomics San Giovanni Rotondo (FG) Italy; ^2^ Department of Experimental Medicine Sapienza University of Rome Rome Italy; ^3^ Department of Biochemical Sciences "A. Rossi Fanelli" Sapienza University of Rome Rome Italy; ^4^ Fondazione IRCCS Casa Sollievo della Sofferenza Laboratory of Cytogenetics San Giovanni Rotondo (FG) Italy; ^5^ Fondazione IRCCS Casa Sollievo della Sofferenza Laboratory of Bioinformatics San Giovanni Rotondo (FG) Italy; ^6^ Fondazione IRCCS Casa Sollievo della Sofferenza Laboratory of Medical Genetics San Giovanni Rotondo (FG) Italy

**Keywords:** Dandy–Walker malformation, fetal imaging, FKTN, muscular dystrophy‐dystroglycanopathy type A4, prenatal diagnosis, whole exome sequencing

## Abstract

**Background:**

Posterior fossa malformations are among the most diagnosed central nervous system (CNS) anomalies detected by ultrasound (US) in prenatal age. We identified the pathogenic gene mutation in a male fetus of 17 weeks of gestation with US suspicion of familial Dandy–Walker spectrum malformation, using Next Generation Sequencing approach in prenatal diagnosis.

**Methods:**

Whole exome sequencing (WES) approach has been performed on fetal genomic DNA. After reads preprocessing, mapping, variant calling, and annotation, a filtering strategy based on allelic frequency, recessive inheritance, and phenotypic ontologies has been applied. A fetal magnetic resonance imaging (MRI) at 18 weeks of gestation has been performed. An in silico analysis of a potential causative missense variant in the fukutin protein has been carried out through a structural modeling approach.

**Results:**

We identified a new homozygous missense mutation in fukutin gene (*FKTN*, NM_006731.2: c.898G>A; NP_006722.2: p.Gly300Arg). Fetal MRI supported molecular findings. Structural modeling analyses indicated a potential pathogenetic mechanism of the variant, through a reduced activation of the sugar moieties, which in turn impairs transfer to dystroglycan and thus its glycosylation. These findings pointed to a redefinition of the US suspicion of recurrence of Dandy–Walker malformation (DWM) to a muscular dystrophy‐dystroglycanopathy type A4.

**Conclusions:**

The present case confirmed WES as a reliable tool for the prenatal identification of the molecular bases of early‐detected CNS malformations.

## Graphical abstract

Prenatal diagnosis of central nervous system (CNS) anomalies following standard diagnostic procedures is challenging. Posterior fossa malformations are among the most diagnosed CNS anomalies detected by ultrasound in prenatal age. Whole exome sequencing (WES) approach on fetal genomic DNA and structural in silico analysis helped redefine a prenatal clinical suspicious of Dandy–Walker malformation to muscular dystrophy dystroglycanopathy Type A through the identification of a new fukutin homozygous missense mutation (NM_006731: c.898G>A; NP_006722.2: p.Gly300Arg). Prenatal diagnosis in cases of nonspecific or early‐detected CNS phenotypes can be lengthy and difficult. Multidisciplinary diagnostic approach combining instrumental (high‐quality fetal magnetic resonance imaging) and molecular analyses (WES) in fetuses with CNS structural anomalies could be a reliable approach.

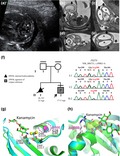

## 1 INTRODUCTION

Prenatal diagnosis of central nervous system (CNS) anomalies following standard diagnostic procedures is challenging, often requiring high‐quality magnetic resonance imaging (MRI) images with sagittal views of the vermis. Posterior fossa malformations are among the most diagnosed CNS anomalies detected by ultrasound (US) in prenatal age. Dandy–Walker malformation (DWM, MIM 220200) is the most common cerebellar malformation, characterized by the evidence of complete or partial agenesis of the cerebellar vermis, cystic dilatation of the fourth ventricle and enlarged posterior fossa with upward displacement of the tentorium, torcula, and transverse sinuses (D'Antonio et al., 2016). Dandy–Walker malformation mainly occurs sporadically, with a low recurrence risk. Isolated DWM is rare, and molecular causes have not been fully recognized. Conversely, several chromosomal and genetic mutations have been identified as causative of syndromes that include this anomaly. Whole exome sequencing (WES) is a powerful tool for the identification of rare diseases causative variants and has proven to be a feasible and effective approach for prenatal diagnosis (Vora et al., 2017).

Here, we report on a new homozygous fukutin (*FKTN*, MIM 607440) missense mutation detected by WES in a fetus with an US suspicion of familial DWM.

## 2 CLINICAL REPORT

**Figure 1:**
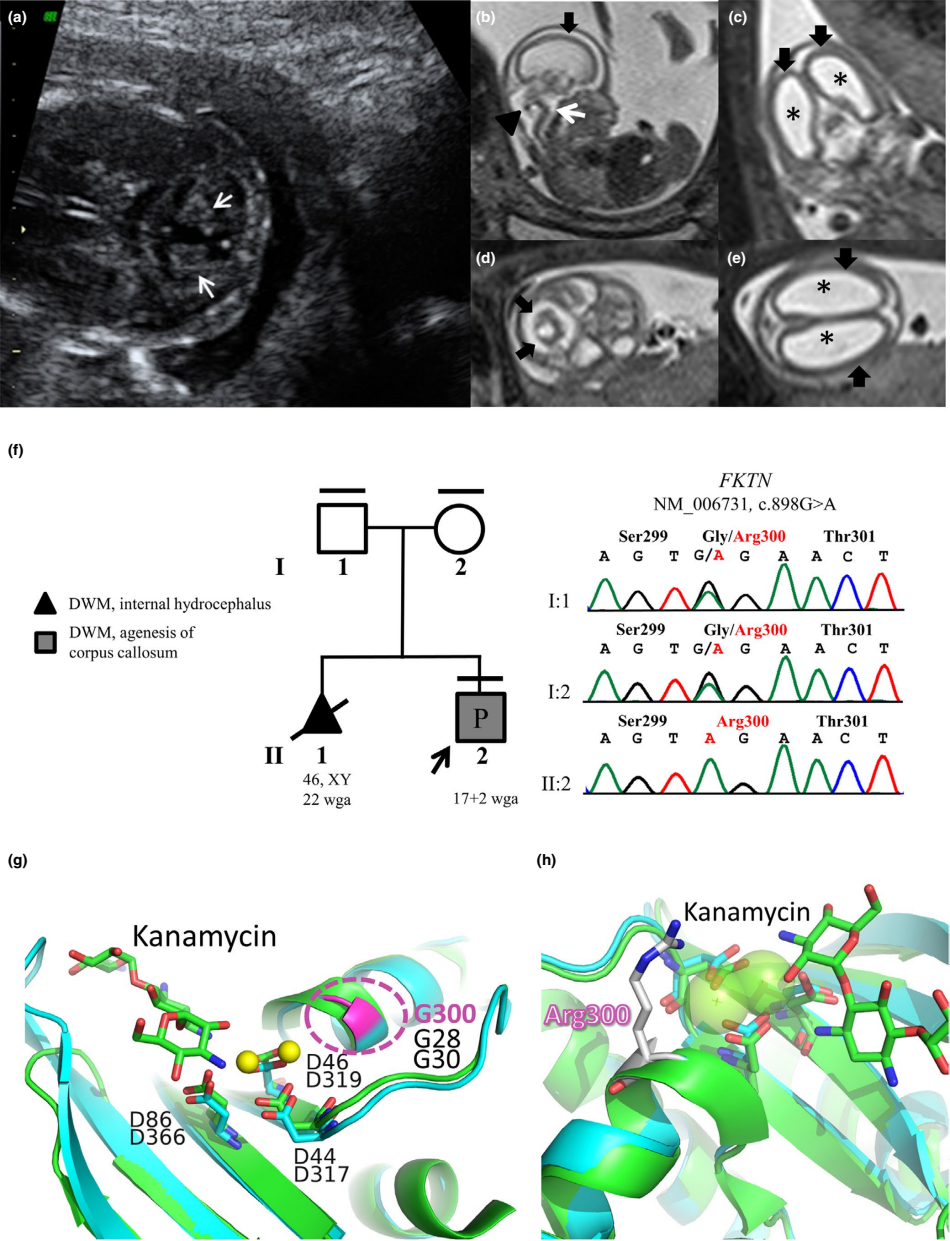
(a) II:2 US imaging (17 + 1 wga). Cerebellar hypoplasia with cerebellar lobes diastasis (white arrows) is visible. (b–e) II:2 Fetal magnetic resonance imaging (18 wga). T2‐weighted images in sagittal (b), coronal (c), and transversal (d, e) planes. The T2 sagittal image (b) shows hypoplasia of the cerebellar vermis (arrowhead) associated with a “Z shaped” brainstem (white arrow) and lissencephaly (black arrow). Coronal and axial planes (c, e) show marked ventriculomegaly (*) and lissencephaly (black arrows). Hypoplasia of the cerebellar hemispheres is visible on transversal plane (d, black arrows). (f) Pedigree of the family and fukutin gene (*FKTN*) chromatograms of the fetus (II:2) and parents (I:1 and I:2). Black lines at the top indicates available DNAs. Black arrow indicates the fetus who underwent WES (II:2, pregnancy [P]). Clinical diagnoses of both fetuses are shown. Sanger analysis confirmed the presence of the homozygous *FKTN* mutation (chr9:108377676‐108377676 [GRCh37/hg19]; NM_006731.2: c.898G>A; NP_006722.2: p.Gly300Arg) in the proband and demonstrated the carrier status of both parents. (g, h) Structural modeling of FKTN variant. (g) Comparison between the homology modeled hFKTN (residues 278‐411; cyan) and one of its structural templates (4WQL, green). The two Mg^2+^ ions are displayed as yellow spheres. Conserved Asp residues in model and X‐ray structure coordinating the ions are shown as sticks and labeled. The aminoglycoside Kanamycin is also shown as green sticks. The position of Gly300 is shown in pink. (h) Detailed view of the active site cleft with modeled the putative position of Arg300

A 34‐year‐old Italian woman was referred to the Prenatal Diagnosis Centre of Umberto I Hospital (Rome, Italy) for a US finding of posterior fossa anomaly in a male fetus at 17 + 2 weeks of gestational age (wga). US examination performed at 17 + 1 wga showed cerebellar hypoplasia, not visible vermis and cerebellar lobes diastasis (Figure 1a) associated with ventriculomegaly and cavum septum pellucidum agenesis. The woman and her partner were healthy, and no consanguinity was referred. They reported a previous pregnancy of a male fetus, interrupted at 22 wga following US findings of a cerebellar malformation in the Dandy–Walker spectrum with a severe internal hydrocephalus. In that case, the fetopsy confirmed the US findings. The diagnosis at the first pregnancy and the US findings of the second pregnancy suggested a recurrence of a phenotype in the DWM spectrum. An amniocentesis was performed at 17 + 4 wga. A single nucleotide polymorphism (SNP) array analysis with an effective resolution of 75 kb (GeneChip 6.0 platform; Affymetrix) on DNA isolated from amniotic fluid resulted negative for copy number variants with likely significant functional impact and demonstrated genome‐wide homozygosity levels of 1% in the fetus, excluding parental consanguinity. As the posterior fossa anomalies in the two male fetuses highly suggested a shared genetic cause of the disease, a WES was performed. Moreover, to clarify the US findings, a fetal MRI (FMRI) including sagittal views of the vermis and T2‐weighted images was requested to evaluate possibly associated intracranial anomalies frequently escaping sonographic diagnosis (D'Antonio et al., 2016).

## 3 METHODS

### 3.1 Ethical compliance

Samples and information were collected after written informed consent was obtained and in accordance with the principles of the Declaration of Helsinki.

### 3.2 Whole exome sequencing

Genomic DNA of the fetus (II:2) was used for a whole exome enrichment and sequencing at CRIBI Genomics (Padua, Italy). Library preparation, amplification, and sequencing, as well as reads preprocessing, mapping against GRCh37/hg19 assembly and variant calling, were performed using the Ion Proton™ kit and sequencing system and the Torrent Suite Software package v5.2.1 (Life Technologies). Variants and genes annotation were performed using ANNOVAR (Wang, Li, & Hakonarson, 2010; July 2017 release) and dbNSFP v3.5 (Liu, Wu, Li, & Boerwinkle, 2016). The identified and functionally annotated single‐nucleotide variants (SNVs) and short insertions and deletions (indels) were filtered to retain only those located in exons with an effect on the coding sequence and splice site regions (±10 bp). The resulting coding and splice site variants were filtered by frequency on public variant database (gnomAD 2.0) to retain only novel/unknown/minor allele frequency (MAF) ≤1% variants. Candidate variants were prioritized considering their predicted functional impact, as evaluated by the Combined Annotation Dependent Depletion (CADD) scoring system v1.3 (Kircher et al., 2014), and the potential or known involvement of the gene in the pathogenesis using a phenotype‐based prioritization tool (Phenolyzer; Yang, Robinson, & Wang, 2015), using the term “Aplasia/Hypoplasia of the cerebellar vermis” (HPO term: 0006817). Variants fitting autosomal and X‐linked recessive inheritance models were firstly considered. The *FKTN* variant validation and segregation analysis were performed through Sanger sequencing (ABI BigDye Terminator Sequencing Kit V.3.1, ABI Prism 3500 Genetic Analyzer). As reference, *FKTN* sequence GenBank NG_008754.1 was used.

### 3.3 Structural modeling of FKTN variant

In order to test the impact of the identified FKTN variant on protein structure and function, a domain analysis and fold recognition search of human FKTN (hFKTN) protein (NP_001073270.1) using Phyre2 server (Kelley, Mezulis, Yates, Wass, & Sternberg, 2015) were carried out.

## 4 RESULTS

Whole exome sequencing data analysis identified a total of 46.610 high‐quality variants (SNVs/indels). Among the 11.302 variants with an effect on coding/splice site regions, filtering strategy based on allele frequency identified 856 novel/unknown/with a MAF ≤ 1% variants.

Ranking of variants assuming an autosomal or X‐linked recessive inheritance model allowed the identification of a homozygous missense mutation in *FKTN* (chr9:108377676‐108377676 [GRCh37/hg19]; NM_006731.2: c.898G>A; NP_006722.2: p.Gly300Arg) as the best candidate. The variant was not annotated in gnomAD 2.0 and was described in dbSNP150 (rs909129168) without reporting any frequency information. The substitution of this highly conserved Gly residue with an Arg (Figure S1) has been predicted as highly deleterious by CADD (score 34) and classified as a “variant of uncertain significance” by InterVar (Li & Wang, 2017). The *FKTN* variant was confirmed and segregation analysis demonstrated the carrier status of both parents (Figure 1f). The segregation analysis could not be performed on fetus of the first pregnancy as no biological sample was available.

As defined by SNP array, the variant mapped within a 1.1 Mb region of homozygosity at 9q31.1q31.2. The identified *FKTN* variant (NM_006731.2, NM_001079802.1, NM_001198963.2: c.898G>A) causes a missense substitution (NP_006722.2, NP_001073270.1, NP_001185892.1: p.Gly300Arg) localized in the sixth or seventh coding exon of *FKTN*, depending on the isoform, which corresponds to a highly conserved region of the protein that acts as a glycosyltransferase.

Concurrently to WES data analysis, FMRI performed at 18 wga showed a marked dilatation of the ventricular system (right ventricle and left ventricle of 13 mm) and a secondary thin cerebral parenchyma. The corpus callosum was absent and the cerebellum was markedly hypoplastic, with a severe reduction of the height of the vermis. A very thin and kinked Z‐shaped brainstem with a flattening of the pons and medulla oblongata was observed (Figure 1b–e). In order to test the pathogenicity of the variant, a structural modeling was performed. Fold recognition on hFKTN (NP_001073270.1) showed that residue Gly300 is part of a nucleotidyltransferase fold comprising residues 278‐411 of hFKTN. In spite of the low sequence identity with the most similar representatives of this fold whose structure is known [seq. id. 12% with ANT(2″)‐Ia (PDB Code: 4WQL) and 16% with LnuA (PDB Code: 4FO1)], the high statistical confidence provided by the server (97.8%), together with the absolute evolutionary conservation of the secondary structure elements (predicted vs. observed in templates) and of the key residues for catalysis, indicate that the Gly300Arg mutation falls in the nucleotidyltransferase fold of hFKTN. In the latter, a cleft of both X‐ray structures (used for homology modeling) harbors two Mg^2+^ ions, which are chelated by Asp_317_, Asp_319_, and Asp_366_ in FKTN (corresponding to the evolutionarily conserved Asp_44_, Asp_46,_ and Asp_86_ of ANT(2″)‐Ia structure; Figure 1g). The 2″‐OH (the site of *O*‐adenylylation) of the sugar substrate, modeled according to the sugar (kanamycin) moiety that is found in the crystal structure of one of the templates (4WQL), is another coordinating site of Mg^2+^ ions.

Comparing the model of hFKTN with the crystal structures of its templates and the well‐studied catalysis of the latter allows the proposal of a mechanism for sugar adenylylation by hFKTN. In this process, binding of the nucleoside triphosphate groups is stabilized by the two Mg^2+^ ions in the active site. Then, the substrate 2″‐OH makes a nucleophilic attack on the α‐phosphate of the nucleoside triphosphate. Catalysis relies therefore on coordination of Mg^2+^ ions, and positioning of the substrate targeted for modification. The nucleotidyltransferase fold of FKTN is thus predicted to activate sugar moieties for subsequent transfer to dystroglycan (DAG1). Replacing Gly300 of FKTN (corresponding to the evolutionarily conserved Gly28 and Gly30 in 4WQL and 4FO1, respectively) with an Arginine residue (Figure 1h) is therefore predicted to have a severe steric hindrance that prevent the nucleotide triphosphate from binding the cleft, thereby abolishing the activation of the sugar moiety with AMP. The latter activity is required for the glycosylation of DAG1 in skeletal muscle; therefore, the damaging effects of Gly300Arg mutation could be explained from a structural point of view as a loss of function of the protein.

Taken together, molecular analyses pointed to a muscular dystrophy‐dystroglycanopathy type A as the final diagnosis. FMRI findings supported molecular results in the diagnosis of muscular dystrophy‐dystroglycanopathy type A, the most severe condition among muscular dystrophy‐dystroglycanopathies. The couple decided to terminate the pregnancy.

## 5 DISCUSSION

Dystroglycanopathies are a group of ~30 autosomal recessive disorders, characterized by muscular dystrophy associated with variable neurological and ophthalmic anomalies. Common mechanism underling dystroglycanopathies is the reduced or absent glycosylation of DAG1, a transmembrane glycoprotein that acts in the dystrophin–glycoprotein complex at the neuromuscular junction and in CNS, connecting the actin associated cytoskeleton to the extracellular matrix in skeletal muscle cells, neurons, and glia. To date, at least fourteen genes have been recognized as causative of dystroglycanopathies: *POMT1*, *FKTN*, *POMGNT1*, *CRPPA*, *DAG1*, *GMPPB*, *POMK*, *RXYLT1*, *POMT2*, *FKRP*, *LARGE1*, *B4GAT1*, *B3GALNT2*, and *POMGNT2*. These genes mostly encode known or putative glycosyltransferases involved in the glycosylation of DAG1. *FKTN* encodes a glycosyltransferase that is ubiquitously expressed, with high levels in cerebellum. It is a putative type II transmembrane protein that is targeted to the Golgi apparatus through an N‐terminal signal sequence (Esapa et al., 2002). Although its function is not completely characterized, FKTN has been recently demonstrated to cooperate with other dystroglycanopathy‐related proteins, such as ISPD, FKRP, and POMT1, for the glycosylation of DAG1 through the incorporation of a ribitol phosphate group in the DAG1 glycan (Gerin et al., 2016; Kanagawa et al., 2016) and is thought to exert a role in brain development (Sudo et al., 2018). Within dystroglycanopathies clinical spectrum (Godfrey et al., 2007), *FKTN* mutations are causative of 3 distinct phenotypes, which differ in clinical severity and CNS involvement. Muscular dystrophy‐dystroglycanopathy type B4 (MIM 613152) is characterized by congenital muscular dystrophy without CNS malformations or cognitive impairment. Muscular dystrophy‐dystroglycanopathy type C4 (MIM 611588), also known as limb‐girdle muscular dystrophy, is mainly defined by proximal muscle weakness, delayed motor development, and muscular dystrophy but no CNS abnormalities. Differently from the other two forms, the most severe muscular dystrophy‐dystroglycanopathy type A4 (MIM 253800) is characterized by structural brain defects and eye abnormalities (i.e., type II lissencephaly, cerebellar and retinal anomalies), and congenital muscular dystrophy. Common associated findings include ventricular dilatation, hydrocephalus, and malformation of the anterior chamber of the eye. Additional features, such as DWM, congenital macrocephaly or microcephaly, microphthalmia, congenital cataracts, and cleft lip palate, occur less frequently.

Besides *FKTN* variants, dystroglycanopathy type A is caused by homozygous or compound heterozygous mutations in all other genes responsible for dystroglycanopathies, and no clear genotype–phenotype correlation has yet been achieved, even if clinical severity seems to inversely correlate with the residual enzymatic activity (Mercuri et al., 2009). For these clinical conditions, a reliable and precocious prenatal diagnosis is difficult to obtain with standard imaging approaches. This is because some signs of these disorders are not highly specific (such as posterior fossa anomalies and cerebellar abnormalities, like DWM, and hydrocephalus), difficult to be early detected with US (i.e., type II lissencephaly and hypoplastic brainstem) or can only be assessed after birth (i.e., congenital muscular dystrophy or retinal abnormalities).

Recently, a prenatal diagnosis of muscular dystrophy‐dystroglycanopathy type A4 has been reported in a fetus of a nonconsanguineous couple of Ashkenazi Jewish with US findings of ventriculomegaly and posterior encephalocele and a known founder pathogenic mutation in *FKTN* (Daum et al., 2018).

These results confirm that antenatal US is a limited approach for the evaluation of posterior fossa anomalies, but it could provide clues to investigate molecular bases of early‐detected CNS malformations, through a combined genetic (i.e., WES) and imaging (FMRI) approach, allowing a final diagnosis in the prenatal period.

In this study, prenatal WES and FMRI have been successfully applied to identify the molecular cause of the disease in a fetus clinically suspected at first as affected by a recurrence of DWM, highlighting the difficulty to identify cerebral malformation at early gestational age. The finding of a new potentially pathogenic homozygous variant p.Gly300Arg in FKTN and the FMRI findings led to a conclusive genetic diagnosis of dystroglycanopathy type A4 to the family, aiding timely management and counseling for pregnancy, and permitting to assess proper recurrence risk for the couple. Moreover, reporting on a new mutation and a putative functional effect can be valuable in understanding pathogenic mechanisms involving FKTN and performing genotype–phenotype correlations in the dystroglycanopathies phenotype spectrum. This work confirms that a reliable prenatal diagnosis in cases of nonspecific or early‐detected CNS phenotypes can be lengthy and difficult and underlies the value of WES approach in fetuses with CNS structural abnormalities following normal karyotype and chromosomal microarray analysis.

## CONFLICTS OF INTEREST

None declared.

## AUTHORS CONTRIBUTION

A. T. contributed to conception, acquisition, and interpretation of data, drafted the manuscript. S. B. contributed to acquisition and interpretation of data, critically revised the manuscript. A. P. contributed to analysis and interpretation of data, drafted the manuscript. A. G., E. M., M. L. G., D. G., B. T., and S. P. contributed to analysis of data and critically revised the manuscript. L. B. and M.C. contributed to interpretation of data and critically revised the manuscript. T. M. contributed to acquisition and analysis of data, critically revised the manuscript. V. C. and A. Pi. contributed to conception, design, acquisition, analysis, interpretation of data, drafted and critically revised the manuscript. All authors approved the final version to be published.
