# Supplementary material for: Prenatal whole exome sequencing detects a new homozygous fukutin (FKTN) mutation in a fetus with an ultrasound suspicion of familial Dandy–Walker malformation
Source: Mol Genet Genomic Med. 2019 Nov 22;8(1):e1054. doi: 10.1002/mgg3.1054 (PMC6978243; doi:10.1002/mgg3.1054)
Supplement: Supplementary file 1 [file MGG3-8-e1054-s001.docx]

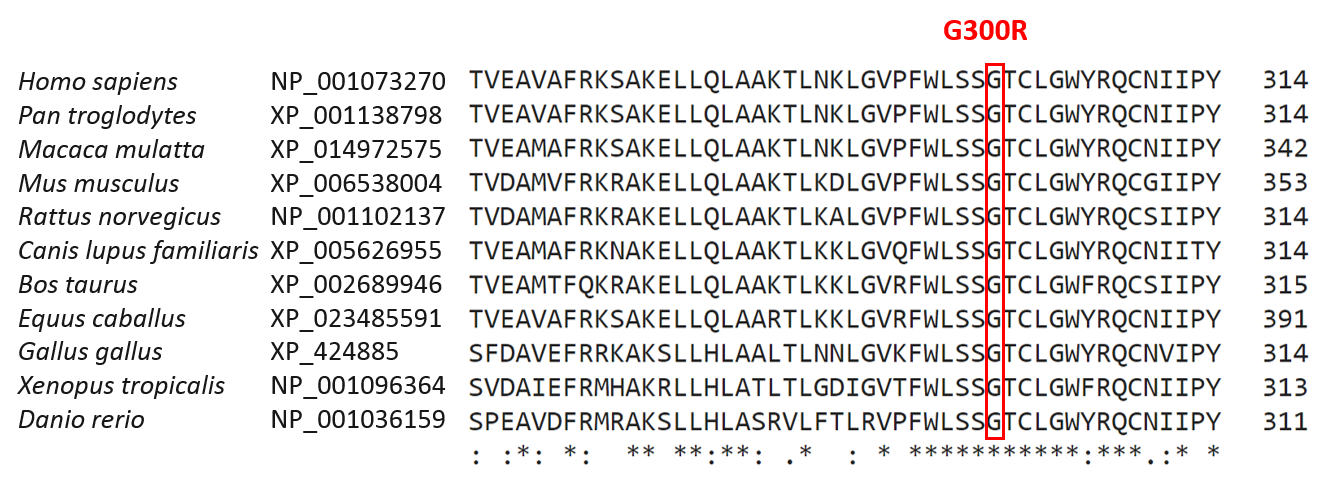


**Figure S1.** Protein alignment shows conservation of residue p.Gly300Arg of FKTN across eleven species. The missense variant occurs at a highly evolutionarily conserved amino acid, in a conserved amino acid sequence. The ClustalW Multiple Sequence Alignments algorithm was used to evaluate sequence conservation (Madeira et al., 2019).

**Reference**

Madeira, F., Park, Y. M., Lee, J., Buso, N., Gur, T., Madhusoodanan, N., … Lopez, R. (2019). The EMBL-EBI search and sequence analysis tools APIs in 2019. *Nucleic Acids Research,* *47*(W1), W636-W641. *doi*:10.1093/nar/gkz268
